# Examining two sets of introgression lines reveals background-independent and stably expressed QTL that improve grain appearance quality in rice (*Oryza sativa* L.)

**DOI:** 10.1007/s00122-017-2862-z

**Published:** 2017-03-15

**Authors:** Xianjin Qiu, Kai Chen, Wenkai Lv, Xiaoxue Ou, Yajun Zhu, Danying Xing, Longwei Yang, Fangjun Fan, Jie Yang, Jianlong Xu, Tianqing Zheng, Zhikang Li

**Affiliations:** 1 Hubei Collaborative Innovation Center for Grain Industry / College of Agriculture, Yangtze University, Jingzhou, 434025 China; 2 Institute of Crop Science, National Key Facility for Crop Gene Resources and Genetic Improvement, Chinese Academy of Agricultural Sciences, Beijing, 100081 China; 3 Agricultural Genomics Institute, Chinese Academy of Agricultural Sciences, Shenzhen, 518120 China; 4 Shenzhen Institute of Breeding for Innovation, Chinese Academy of Agricultural Sciences, Shenzhen, 518120 China; 5 Institute of Food Crops, Jiangsu High Quality Rice Research and Development Center, Nanjing Branch of China National Center for Rice Improvement, Jiangsu Academy of Agricultural Sciences, Nanjing, 210014 China

## Abstract

***Key message*:**

**A novel QTL cluster for appearance quality on Chr07 was identified using reciprocal introgression populations in different locations in China. Two secondary F**_**2**_ **populations validated QTL with significant effect on appearance quality**.

**Abstract:**

Appearance quality (AQ) is the main determinants of market value of rice. Identification of QTL affecting AQ is the prerequisite for efficient improvement of AQ through marker-assisted selection (MAS). Two sets of reciprocal introgression lines derived from *indica* Minghui 63 and *japonica* 02428 were used to dissect the stability of QTL affecting five AQ traits, including grain length, grain width, length to width ratio, percentage of grains with chalkiness, and degree of endosperm chalkiness using 4568 bin genotype produced from 58,000 SNPs across five different environments. A total of 41 and 30 main-effect QTL were identified in MH63 and 02428 backgrounds, respectively. Among them, 9 background-independent QTL (BI-QTL) were found. There were also 13 and 10 stable-expressed QTL (SE-QTL) across at least two environments in MH63 and 02428 backgrounds, respectively. Two important BI- and SE-QTL regions (BISERs) including BISER-I harboring *qPGWC5, qDEC5, qGW5.1*, and *qLWR5* on chromosome 5 and BISER-II harboring *qGL7, qLWR7, qPGWC7*, and *qDEC7* on chromosome 7 were identified. The BISER-II was newly reported and validated by two secondary F_2_ populations in the reciprocal backgrounds. Among 59 epistatic QTL (E-QTL) detected in this study, there were only four SE- but no BI-E-QTL detected in different environments, indicating that genetic background has stronger effect on AQ traits than the environmental factors, especially for percentage of grains with chalkiness (PGWC) and degree of endosperm chalkiness (DEC) with lower heritability. BISER-I and BISER-II harboring many BI- and SE-QTL with favorable alleles from slender grain rice are much important for improvement of rice AQ by MAS.

**Electronic supplementary material:**

The online version of this article (doi:10.1007/s00122-017-2862-z) contains supplementary material, which is available to authorized users.

## Introduction

Rice (*Oryza sativa*. L) is one of the most important crops in the world, providing a carbohydrate source for half of the world’s population. With the economy development of rice-consuming area, grain quality especially the appearance quality (AQ) is being attracted more attention by both consumers and producers than ever before. It has become one key target trait equivalent to the grain yield in the rice breeding program (Tan et al. 2000). Rice AQ consists of grain shape and grain chalkiness. Grain shape is composed of grain length (GL), grain width (GW), and length to width ratio (LWR). Different shapes of grains (de-hulled seeds) have different market values in different areas (Luo et al. 2004). Most people in Southern China, USA, Southern and Southeast Asia prefer long and slender grains, whereas short and round ones are preferred by people in Northern China, Japan, and Korea (Juliano and Villareal 1993; Unnevehr et al. 1992). Besides, grain shape is one of determination factors for grain weight. Grain chalkiness, including white-back, white-core, and white-belly kernels based on the different parts of the grain that are chalky (Li et al. 2004; Satoh and Omura 1981; Tan et al. 2000), is an undesirable grain character. It is easy to cause the broken grains during milling, and can even affect palatability of cooked rice (Cheng et al. 2005; del Rosario et al. 1968; Nagato and Ebata 1959). Percentage of grains with chalkiness (PGWC) and degree of endosperm chalkiness (DEC) are two standards commonly used to evaluate grain chalkiness. Nowadays, AQ traits have become more and more important in breeding schemes in rice producing areas around the world, especially for hybrid rice in China (Tan et al. 2000).

All AQ traits are typically quantitative traits controlled by multiple genes and affected by environmental factors (He et al. 1999; Tan et al. 2000; Yamakawa et al. 2007). A large number of QTL for GL, GW, LWR, PGWC, and DEC, located on all 12 chromosomes, have been reported in various mapping populations in rice. Among them, *GS2* (Zhang et al. 2013), *qGRL1.1* and *qGRL7.1* (Singh et al. 2012), *qGL7* (Bai et al. 2010), *qSS7* (Qiu et al. 2012) for grain shape, *qPGWC-7* (Zhou et al. 2009), *qPGWC-8* (Guo et al. 2011) for grain chalkiness have been fine mapped. And *GS3* (Fan et al. 2006; Mao et al. 2010), *GS2*/*GL2* (Che et al. 2015; Hu et al. 2015), *GL3.1* (Qi et al. 2012; Zhang et al. 2012), *GL7*/*GW7* (Wang et al. 2015a, b), *GW2* (Song et al. 2007) and *qSW5*/*GW5* (Shomura et al. 2008; Weng et al. 2008), *GS5* (Li et al. 2011) and *GW8* (Wang et al. 2012) have been cloned. It seems that *GS3, GW2, qSW5*/*GW5, GL3.1, GS2*/*GL2* are negative regulators of grain shape, while *GS5, GW8, GL7*/*GW7* are positive regulators.

Some genes affecting grain chalkiness have been identified. *OsPPDKB*, encoding pyruvate orthophosphate kinase (PPDK), contributes to the control of carbon flow into starch and lipid biosynthesis during grain filling (Kang et al. 2005). *SSIIIa*, encoding starch synthase IIIa, plays an important role in the elongation in amylopectin chains (Fujita et al. 2007; Ryoo et al. 2007). *GW2*, encoding a RING-type E3 ubiquitin ligase, also controls grain chalkiness by grain filling (Song et al. 2007). *GIF1*, encoding a cell-wall invertase, was required for carbon partitioning during grain filling (Wang et al. 2008). *ms-h* gene encodes the UDP-glucose pyrophosphorylase 1 (Woo et al. 2008). *FLO2*, harboring a tetratricopeptide repeat motif and belonging to a novel gene family conserved in plants, was considered to mediate a protein–protein interaction (She et al. 2010). *OsRab5a*, encoding a small GTPase, plays an essential role in trafficking of storage protein to protein body II (Wang et al. 2010). Loss of function in these genes leads to loosely and small granules and increased chalkiness. *Chalk5* encodes a vacuolar H^+^-translocating pyrophosphatase with inorganic pyrophosphate hydrolysis (Li et al. 2014). Elevating its expression increases the chalkiness of the endosperm. Two consensus nucleotide polymorphisms in the *Chalk5* promoter were associated with grain chalkiness. However, even *Chalk 5* can only explain partial (about 30%) of the variances (Li et al. 2014), more new loci are still waiting for mining.

Most QTL/genes mentioned above can be used for marker-assisted selection for improving AQ, but expressions of these QTL are strongly affected by genetic background and environment (Wan et al. 2005; Zhao et al. 2016; Zheng et al. 2011). Strong genetic background effects on grain shape were detected using a set of reciprocal introgression lines from Lemont and Teqing (Zheng et al. 2011). In another report, 22 QTL were identified for rice grain dimension and endosperm chalkiness characteristics in eight environments by a chromosome segment substitution line (CSSL) population from Asomironi and IR24, in which nine QTL were detected in all environments (Wan et al. 2005). Recently, a new research detected 78 and 43 QTL for grain chalkiness by two sets of RILs from reciprocal crosses between Lemont and Teqing (Zhao et al. 2016). Only 14 and 5 QTL were stably expressed across different environments. These problems will probably cause the reduction in the efficiency of molecular breeding to improve rice AQ by the identified QTL.

Although much QTL analysis on AQ has been reported, the genetic background and environment effects on QTL expression was relatively less reported. In the present study, two sets of reciprocal introgression lines (ILs) derived from Minghui 63 (MH63) and 02428 with high density of bin map were used, and the AQ traits were evaluated across five environments. The objectives of this study were to (1) dissect the genetic basis of stability of rice AQ traits, including the identification of more genetic BI- and/or SE-QTL regions for AQ traits and the digenic epistatic QTL for these traits and (2) validate important novel BI- and SE-QTL regions for AQ traits.

## Materials and methods

### Development of reciprocal introgression lines

Two sets of reciprocal ILs were developed from a cross between Minghui 63 (abbreviated as MH63), an elite *indica* restorer parent of the widely adapted hybrid variety Shanyou 63 with slender and low chalky grains, and 02428, a wide compatible *temperate japonica* variety with round and high chalky grains. The F_1_ hybrids were simultaneously backcrossed to MH63 and 02428 to produce the BC_1_F_1_ generation, respectively. The BC_1_F_1_ individuals were then backcrossed with corresponding parents to produce the BC_2_F_1_. The BC_2_F_1_ individuals were selfed for seven generations followed single seed descent method and arrived at BC_2_F_8_ generation. Ultimately, two sets of reciprocal ILs were successfully developed after removal of lines with heading date too late for QTL detection. The reciprocal ILs consists of 226 lines in MH63 background (MH63-ILs) and 198 lines in 02428 background (02428-ILs).

### Field experiment and trait measurement

A total of the 424 reciprocal ILs and parents, MH63 and 02428, were grown in five representing locations in the south of China. There are three locations in the *indica*/*japonica* mix-cultivating area including Jingzhou (JZ, 30.18°N, 112.15°E) in the middle stream of the Yangtze River, and Nanjing (NJ, 32.03°N, 118.46°E) and Xuzhou (XZ, 34.15°N, 117.11°E) in the down stream of the Yangtze River. Another two locations were set in the two-season *indica* cultivating area of southern China, including Shenzhen (SZ, 22.33°N, 114.07°E) and Sanya (SY, 18.31°N, 108.56°E). Field tests were conducted using a randomized complete block design with two replications. The seeding and transplanting at each location were following the normal cultivating arrangement in major farming season, including a winter season at SY. At each location, reciprocal ILs and their parents were planted in three-row plots with ten individuals in each row at spacing of 20 cm × 20 cm. All field managements followed local farmers’ practices. At maturing stage, eight individuals in the middle row of each line were harvest in bulk. After natural drying, grains were stored at room temperature for at least 3 months for trait measurement.

The GL (mm) and GW (mm) were measured according to the National Rice Grain Quality Assessment Standard of China (GB/T17891-1999). PGWC (%) and DEC (%) were measured using a rice appearance quality detector (Dong Fu Jiu Heng, JMWT12, Beijing). LWR was the ratio of GL to GW. PGWC was the percentage of head milled grains with chalkiness. DEC was calculated as the product of PGWC and chalk size, which was the area of chalk divided by the area of whole grain. All measurements were repeated twice for each sample, and the averaged values were used for data analysis.

### DNA extraction, SNP genotyping, and bin map construction

Young leaves of about the eight plants in the middle row per line were bulk-harvested for DNA extraction. Genomic DNA of the two parents and the two sets of ILs were extracted using a DNeasy mini Kit (Qiagen). The genotypes of the ILs were determined based on SNPs generated by whole-genome sequencing with the Illumina Genome Analyzer IIx as described previously (Huang et al. 2009).

MH63 and 02428 were subjected to whole-genome resequencing and a total of 5,336,108,154 and 5,562,905,674 nucleotides of data were obtained. Alignment was performed against the Nipponbare sequence (IRGSP 1.0) as the reference genome (Kawahara et al. 2013). 5,062,106,567 and 5,278,080,725 nucleotides were obtained for MH63 and 02428, covering 96.57 and 94.03% of the whole genome, respectively. After that 58,936 SNPs were found between MH63 and 02428. Finally, a bin map containing 4568 bins was constructed in the two ILs based on these SNPs as described before (Xie et al. 2010).

### Data analysis

Correlations analysis and the analysis of variance (ANOVA) were carried out by Statistica 5.5 (StaSoft 1999). The broad-sense heritability (*h* ^2^) was calculated based on the routing method (Hallauer et al. 2010).

Main-effect QTL (M-QTL) and digenic epistatic QTL (E-QTL) were detected by using the inclusive interval mapping (ICIM) function with bi-parental population (BIP) module in QTL IciMapping ver. 4.0 (Li et al. 2007). LOD thresholds for M-QTL detection were determined by 1000 permutation tests as listed in Table S1 with averaged LOD values of 2.8 and 3.3 in MH63-ILs and 02428-ILs, respectively (Churchill and Doerge 1994). M-QTL detected in different environments for the same trait with overlapping confidence intervals was treated as the same locus. E-QTL were claimed under a default threshold of LOD = 3.5.

### Validation of novel important BI- and SE-QTL clusters

The region of 4.8–5.2 Mb on chromosome 7 was detected affecting almost all AQ traits across different environments at both genetic backgrounds. To confirm this region, two ILs, DQ28 and DQ438 were selected from MH63-ILs and 02428-ILs, respectively (Fig. S1), were selected based on the recurrent parents’ genome to backcross with the recurrent parents to produce F_2_ populations. The two segregating populations and parents were planted at Xuzhou; each containing about 200 plants, and were genotyped using five randomly selected SSR markers within the candidate region (Table S4). All individuals with two homozygous genotypes were measured, their AQ traits following the same procedure mentioned above. Further, Duncan *t* test was used to test the differences between different genotypes under a threshold of *P* ≤ 0.01.

## Results

### Bin map of the reciprocal ILs

**Fig. 1:**
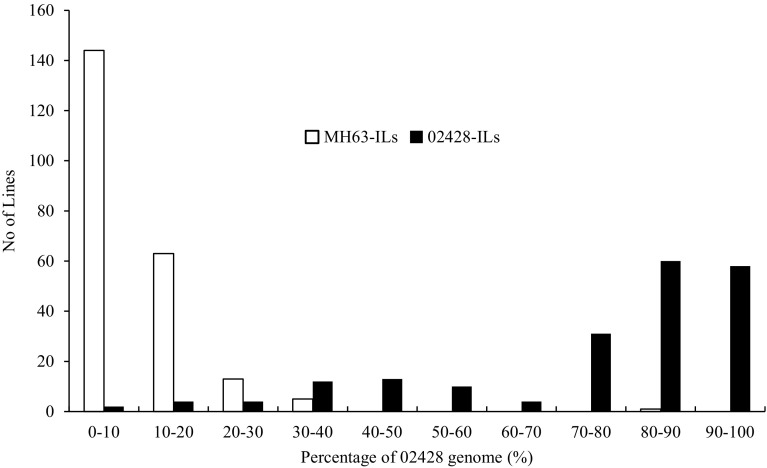
Frequency distribution of 02428 genome in the reciprocal ILs derived from MH63 × 02428

A total of 4568 bins were evenly distributed across 12 chromosomes covering 97.7% (373.24 Mb) of the rice genome published by International Rice Genome Sequencing Project (Kawahara et al. 2013), with average length of 81.71 kb and ranging from 30.0 to 1809.8 kb. Most of the ILs possessed well-reconstituted parental genotypes. The averaged introgression frequencies of MH63-ILs was 8.8% ranged by 0.02–87.6%, whereas the frequencies of 02428-ILs was averagely 23.4%, ranging from 0.64 to 95.1% (Fig. 1).

### Phenotypic performances of reciprocal ILs and their parents

As shown in Table 1, MH63 has more slender grain with lower chalkiness than 02428. This was supported by the significantly higher values of GL (averagely 9.8 mm for MH63 while 7.1 mm for 02428) and LWR (averagely 3.5 for MH63 while 2.1 for 02428) but lower values of GW (averagely 2.9 for MH63 while 3.4 for 02428), PGWC (averagely 9.2 for MH63 while 79.6 for 02428) and DEC (averagely 2.4 for MH63 while 53.8 for 02428) across the five testing locations. The ILs progenies presented phenotypic trends of their recurrent parents. The mean values across the five locations for GL and LWR were 9.6 mm and 3.4 in the MH63-ILs but only 7.5 mm and 2.3 in the 02428-ILs, respectively. As for the GW, PGWC, and DEC, the averaged values of 2.9 mm, 18.5%, and 5.8% were detected for the MH63-ILs but the ones of 3.3 mm, 74.9%, and 46.5% were detected for the 02428-ILs. Transgressive segregations were also observed for all AQ traits in the reciprocal ILs across all the five locations. It is also notable that the PGWC and DEC showed much larger variations than the grain shape traits (Table 1).

**Table 1:** Performances of appearance quality traits of the reciprocal ILs and their parents MH63 (P_1_) and 02428 (P_2_) in five locations

Trait	Loc	Parents			MH63-ILs			02428-ILs		
		P_1_	P_2_	P_1_–P_2_^a^	Mean ± SD	Range	CV (%)	Mean ± SD	Range	CV (%)
GL (mm)	SZ	10.02	7.26	2.76^***^	9.93 ± 0.47	7.69–11.26	4.8	7.80 ± 0.61	6.68–10.07	7.8
	NJ	9.93	7.25	2.67^***^	9.80 ± 0.57	7.30–10.93	5.8	7.59 ± 0.60	6.52–9.73	8.0
	XZ	9.96	7.18	2.78^***^	9.83 ± 0.57	7.15–10.92	5.8	7.58 ± 0.60	6.63–10.09	8.0
	JZ	9.84	7.14	2.70^***^	9.64 ± 0.47	7.80–10.71	4.8	7.50 ± 0.64	6.19–9.63	8.5
	SY	9.21	6.54	2.67^***^	9.03 ± 0.45	6.68–10.05	5.0	6.84 ± 0.57	5.83–9.06	8.3
GW (mm)	SZ	2.97	3.56	−0.59^**^	3.02 ± 0.13	2.75–3.54	4.4	3.49 ± 0.19	2.52–3.78	5.5
	NJ	2.88	3.38	−0.50^**^	2.92 ± 0.15	2.54–3.52	5.1	3.28 ± 0.21	2.39–3.76	6.3
	XZ	2.96	3.41	−0.45^**^	3.02 ± 0.14	2.73–3.65	4.6	3.37 ± 0.20	2.57–3.77	5.8
	JZ	2.73	3.32	−0.59^**^	2.79 ± 0.12	2.44–3.17	4.4	3.25 ± 0.21	2.28–3.70	6.4
	SY	2.59	3.27	−0.67^**^	2.65 ± 0.12	2.43–3.19	4.6	3.20 ± 0.21	2.27–3.53	6.6
LWR	SZ	3.4	2.0	1.3^***^	3.3 ± 0.2	2.3–3.9	6.5	2.3 ± 0.3	1.9–3.7	11.9
	NJ	3.5	2.1	1.3^***^	3.4 ± 0.3	2.1–4.0	8.0	2.3 ± 0.3	1.9–3.7	12.3
	XZ	3.4	2.1	1.3^***^	3.3 ± 0.3	2.0–3.7	7.7	2.3 ± 0.3	1.9–3.4	12.0
	JZ	3.6	2.2	1.4^***^	3.5 ± 0.2	2.7–4.1	7.0	2.3 ± 0.3	1.9–3.8	13.1
	SY	3.6	2.0	1.5^***^	3.4 ± 0.2	2.2–3.9	7.1	2.1 ± 0.3	1.4–3.8	14.6
PGWC (%)	SZ	5.9	82.6	−76.7^***^	14.8 ± 15.6	0.0–79.6	105.2	82.4 ± 20.9	9.5–100.0	25.4
	NJ	5.9	79.6	−73.7^***^	14.8 ± 16.5	0.4–96.1	111.6	69.4 ± 23.0	3.4–100.0	33.2
	XZ	11.3	74.1	−62.8^***^	24.9 ± 19.2	1.7–78.9	77.2	84.8 ± 19.5	7.7–100.0	23.0
	JZ	9.0	79.8	−70.8^***^	12.9 ± 9.9	0.3–61.0	76.9	67.9 ± 23.8	3.6–100.0	35.0
	SY	13.8	81.9	−68.1^***^	25.3 ± 16.5	0.5–80.0	65.1	69.9 ± 23.3	1.0–100.0	33.3
DEC (%)	SZ	1.4	55.9	−54.5^***^	4.2 ± 5.8	0.0–38.3	136.4	55.4 ± 19.5	2.2–85.7	35.2
	NJ	1.3	42.1	−40.8^***^	4.2 ± 7.3	0.1–59.9	173.2	33.2 ± 16.0	1.0–68.2	48.1
	XZ	2.2	59.5	−57.3^***^	7.3 ± 8.7	0.3–57.7	120.2	64.7 ± 21.1	2.3–88.8	32.6
	JZ	2.5	50.2	−47.7^***^	4.0 ± 3.7	0.0–25.3	93.4	38.1 ± 19.0	0.9–77.0	49.9
	SY	4.4	61.4	−57.0^***^	9.2 ± 7.8	0.1–51.8	84.7	41.0 ± 19.3	0.4–83.6	47.1

*GL* grain length, *GW* grain width, *LWR* length to width ratio, *PGWC* percentage of grains with chalkiness, *DEC* degree of endosperm chalkiness

*SZ* Shenzhen, *NJ* Nanjing, *XZ* Xuzhou, *JZ* Jingzhou, *SY* Sanya

^a^*, **, ***The significant level of *P* ≤ 0.05, 0.01, and 0.001, respectively

Correlation coefficients between different traits in the reciprocal ILs are listed in Table S2. The trait correlations were all extremely significant (*P* ≤ 0.001), except for the correlation between GL and GW at SZ (−0.08 with *P* ≤ 0.5) in MH63 background. PGWC was highly positively correlated with DEC. They were negatively correlated with the GL and LWR but strikingly positively related with the GW indicating that slenderer grains had lower chalkiness. Across the five locations, the coefficients between GW and chalkiness were higher than that between GL and chalkiness, indicating that GW has much stronger effect than the GL on the occurrence of chalkiness.

Correlation coefficients between different environments for each trait are given in Table S3. For all traits in the reciprocal ILs, correlations among different environments were significant. Most correlation coefficients among different environments for GL, GW, and LWR were higher than 0.6, whereas that for PGWC and DEC were lower than 0.6, indicating that chalkiness was really affected more by environment than grain shape.

For all traits in the reciprocal ILs, except LWR for *G* × *E* in MH63-ILs, the ANOVA showed genotypes, environments, and the interaction between genotype and environment were all highly significant (Table 2). The broad-sense heritability values, calculated by partitioning the variance into genetic and genotype by environment effects, were above 70% for all traits except LWR in MH63-ILs (53.7%).

**Table 2:** Analysis of variations (ANOVA) for appearance quality traits of the reciprocal ILs across five locations

Trait	Population	Genotype (*G*)^a^	Location (*E*)	*G* × *E*	MSE	Heritability (*h* ^2^, %)
GL	MH63-ILs	41.9^***^	1200.8^*** c^	2.7^***^	0.05	91.9
	02428-ILs	37.6^***^	786.8^***^	1.9^***^	0.08	92.9
GW	MH63-ILs	14.2^***^	1269.3^***^	1.9^***^	0.01	81.3
	02428-ILs	36.9^***^	632.6^***^	2.3^***^	0.01	91.7
LWR	MH63-ILs	2.4^***^	14.6^***^	1.1	0.31	53.7
	02428-ILs	71.9^***^	249.5^***^	3.0^***^	0.01	94.7
PGWC	MH63-ILs	13.2^***^	143.1^***^	2.1^***^	117.0	81.2
	02428-ILs	7.0^***^	82.9^***^	1.7^***^	356.3	72.2
DEC	MH63-ILs	11.4^***^	106.3^***^	2.0^***^	24.9	79.2
	02428-ILs	11.3^***^	436.5^***^	2.3^***^	177.4	77.6

*GL* grain length, *GW* grain width, *LWR* length to width ratio, *PGWC* percentage of grains with chalkiness, *DEC* degree of endosperm chalkiness

^a^ *F* values with *, **, and *** indicating the significant level of *P* ≤ 0.05, 0.01, and 0.001, respectively

### Analysis of M-QTL for AQ traits

**Fig. 2:**
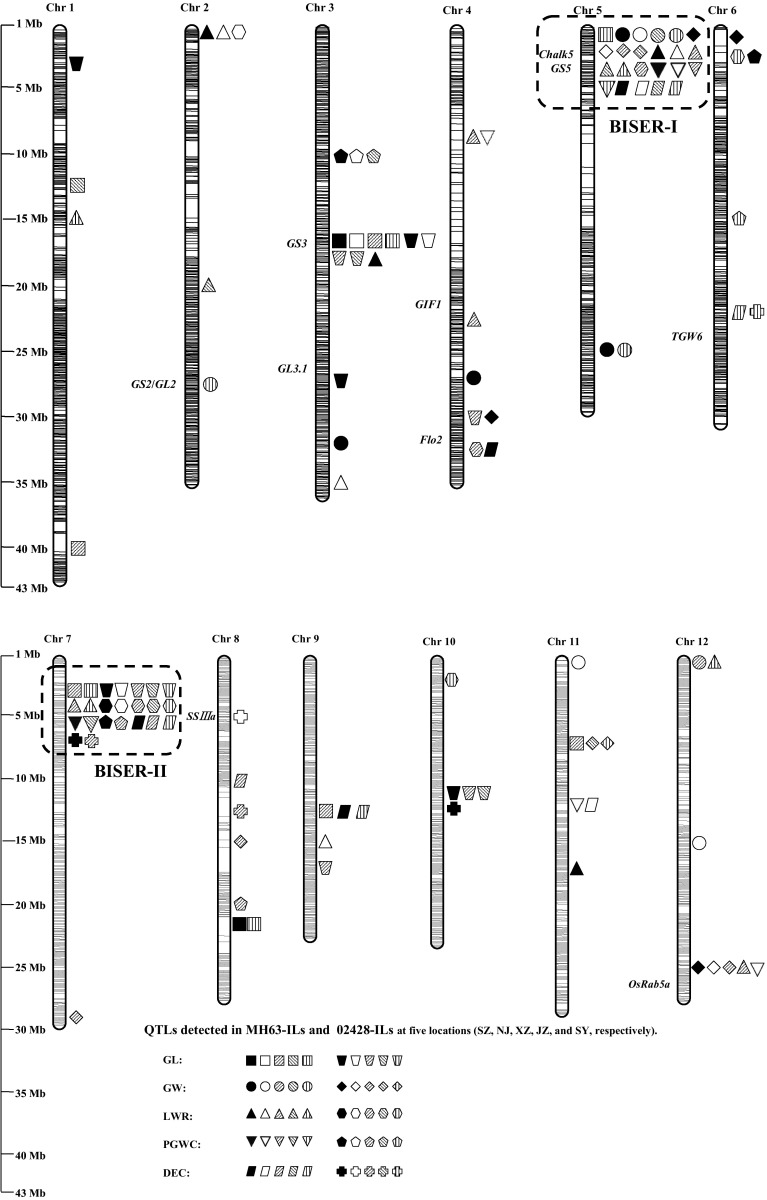
Genome distribution of M-QTL detected in the reciprocal ILs derived from MH63 × 02428 for appearance quality (AQ) traits at five locations. The background-independent and stably expressed QTL regions (BISERs) for AQ were also circled by *dot lines*

Based on bin map, a total of 41 QTL for five AQ traits were identified in MH63-ILs (Table 3; Fig. 2). A total of 8, 8, 13, 5, and 7 QTL were detected on all chromosomes except 10 for GL, GW, LWR, PGWC, and DEC, respectively, in MH63-ILs. The 02428 alleles decreased GL and LWR, and increased GW at all loci, except for *qGL1.2, qGL1.3, qLWR1, qGW4.1, qGL8, qLWR9, qGW11.1*, and *qLWR11*, and were associated with increased PGWC and DEC at all QTL. Among the loci detected in MH63-ILs, 13 (31.7%) QTL distributing on chromosomes 2, 3, 5, 7, 8, and 9 were found to be expressed at no less than two locations. There were five QTL (*qGL3.1, qGW5.1, qLWR5, qPGWC5*, and *qDEC5*) detected in four or five locations with averaged percentage of phenotypic variances explained of 13.4, 36.6, 20.7, 21.3, and 20.8%, respectively. The *qDEC7* was detected at three locations and explained 1.1–14.1% of phenotypic variances. Seven QTL (*qLWR2.1, qGW5.2, qGL7, qLWR7, qPGWC7, qGL8*, and *qDEC9*) were detected at two locations.

**Table 3:** Main-effect QTL (M-QTL) affecting appearance quality traits in the reciprocal ILs derived from MH63 × 02428 across five locations

ILs	Trait	M-QTL	Chr.	Position (Mb)	SZ	NJ	XZ	JZ	SY	Reported genes
MH63	GL	qGL1.2	1	12.4–12.7				4.5/0.12/3.2		
		qGL1.3	1	39.5–40.2			4.4/0.13/2.5			–
		qGL3.1	3	16.2–17.2	43.7/−0.58/25.5	23.6/−0.88/10.3	21.2/−0.51/16.0		3.0/−0.10/1.9	GS3
		qGL5	5	3.4–3.5					9.3/−0.17/4.7	Chalk5, GS5
		qGL7	7	4.8–5.2			6.0/−0.26/8.3		12.0/−0.23/5.8	
		qGL8	8	20.9–23.1	5.8/0.13/2.3				8.3/0.14/3.9	
		qGL9.1	9	11.8–14.0		21.0/−1.02/55.6				
		qGL11	11	11.0–15.5			15.8/−0.26/9.7			
	GW	qGW2	2	27.1–27.8					2.9/0.04/2.5	GS2/GL2
		qGW3	3	31.8–31.9	7.8/0.06/6.2					
		qGW4.1	4	27.0–27.9	3.4/−0.09/2.9					
		qGW5.1	5	3.4–3.5	45.1/0.15/44.1	27.5/0.13/22.9		23.9/0.13/35.9	47.0/0.14/43.3	Chalk5, GS5
		qGW5.2	5	24.8–25.2	8.4/0.08/14.5				6.7/0.05/6.7	
		qGW11.1	11	0.0–0.3		3.6/−0.03/2.9				
		qGW12.1	12	0.0–0.3			2.9/0.04/2.1			
		qGW12.2	12	14.2–17.0		4.9/0.09/6.3				
	LWR	qLWR1	1	14.6–15.4					11.2/0.1/7.3	
		qLWR2.1	2	0.0–0.4	4.7/−0.04/1.8	3.9/−0.1/1.4				
		qLWR2.2	2	19.9–20.5				3.1/−0.2/6.4		
		qLWR3.1	3	16.2–17.2	18.1/−0.1/8.6					
		qLWR3.2	3	34.9–35.4		8.5/−0.2/4.4				
		qLWR4.1	4	7.2–11.4			3.2/−0.2/4.5			
		qLWR4.2	4	22.4–22.6			4.3/−0.1/2.2			GIF1
		qLWR5	5	3.4–3.5	42.7/−0.2/25.5	30.1/−0.2/15.1	23.8/−0.2/17.0	15.3/−0.2/18.6	34.0/−0.2/27.4	Chalk5, GS5
		qLWR7	7	4.8–5.2			11.5/−0.1/6.4		8.3/−0.2/7.5	
		qLWR9	9	14.8–15.1		7.7/0.1/3.2				
		qLWR11	11	17.1–17.8	4.9/0.1/2.2					
		qLWR12.1	12	0.0–0.3					4.6/−0.1/2.6	
		qLWR12.2	12	24.7–25.4			3.8/−0.2/5.1			
	PGWC	qPGWC4	4	7.2–11.4		4.5/19.6/15.0				
		qPGWC5	5	3.4–3.5	22.2/13.8/25.2	9.3/10.1/12.7		24.0/9.6/30.9	13.5/11.7/16.2	Chalk5/qPGWC5a/qPGC5–1/qPGWC5
		qPGWC7	7	4.8–5.2	6.4/12.7/23.1		20.6/6.8/6.4			
		qPGWC11	11	10.7–15.5		6.2/17.0/16.9				
		qPGWC12	12	24.7–25.4		11.7/33.7/15.4				OsRab5a
	DEC	qDEC4	4	27.0–27.9	5.1/5.6/7.4					qWB4
		qDEC5	5	3.4–3.5	24.3/5.6/31.3	14.9/3.3/7.0		30.4/4.2/39.4	4.8/3.2/5.4	Chalk5/qDEC5a/qPGC5-1/qDEC5
		qDEC6	6	22.4–22.8					4.2/3.9/10.1	
		qDEC7	7	4.8–5.2	12.5/3.6/14.1		5.7/5.2/1.1		4.6/2.8/4.7	
		qDEC8.2	8	9.8–10.2			41.9/10.4/25.5			qPGC8-1
		qDEC9	9	11.8–14.0	6.0/14.3/10.9				12.5/8.9/20.0	qDEC9
		qDEC11	11	10.7–15.5		5.4/4.4/4.1				
02428	GL	qGL1.1	1	3.3–3.8	9.4/0.40/15.5					
		qGL3.1	3	16.2–17.2	7.5/0.30/12.6	6.2/0.30/13.9	4.3/0.20/9.8	3.9/0.20/9.1		GS3
		qGL3.2	3	28.0–28.1	5.3/0.20/10.1					GL3.1
		qGL4	4	29.9–30.2			4.3/−0.30/12.6			
		qGL7	7	4.8–5.2	5.6/0.20/5.8	5.4/0.20/12.3	4.9/0.20/11.4	5.4/0.30/12.5	5.2/0.20/9.9	
		qGL9.2	9	17.5–17.6		4.3/−0.30/10.3				
		qGL10	10	12.0–12.2	4.2/0.20/5.1		4.9/0.30/12.8	5.2/0.30/13.0		
	GW	qGW4.2	4	29.9–30.2	3.9/−0.10/6.2					Flo2
		qGW5.1	5	3.4–3.5	4.6/−0.10/8.6	4.2/−0.10/9.7	5.1/−0.10/11.5	3.1/−0.10/7.3		Chalk5, GS5
		qGW6	6	0.3–2.4	4.1/−0.10/5.2					
		qGW7	7	29.1–29.4			4.3/−0.10/8.9			
		qGW8	8	14.9–17.4			5.1/−0.10/8.6			
		qGW11.2	11	11.7–12.7				6.3/−0.10/14.1	5.2/−0.10/12.1	
		qGW12.3	12	24.8–25.2		18.9/−0.10/39.1	6.7/−0.10/11.2	5.1/−0.10/10.1		
	LWR	qLWR2.1	2	0.0–0.2		3.7/0.1/8.0				
		qLWR4.3	4	32.3–32.6		5.4/0.1/9.7				Flo2
		qLWR5	5	3.4–3.5		4.6/0.1/10.5				Chalk5, GS5
		qLWR6	6	2.4–3.2					3.5/0.1/6.6	
		qLWR7	7	4.8–5.2	3.8/0.1/7.8	5.5/0.1/12.5	5.9/0.1/13.4	5.2/0.1/11.7	4.2/0.1/7.5	
		qLWR10	10	2.3–2.6				4.4/0.1/10.1		
	PGWC	qPGWC3	3	10.0–10.1	5.9/−9.1/13.7	3.5/−7.5/6.7		4.8/−8.7/11.1		
		qPGWC6.1	6	2.4–3.2	4.3/−8.4/10.1					qPGWC6
		qPGWC6.2	6	15.0–15.2					3.2/−7.7/7.5	
		qPGWC7	7	4.8–5.2	5.3/−7.2/11.5		4.9/−6.8/8.1			
		qPGWC8	8	19.9–20.3			3.4/−7.6/8.8			qPGWC8b
	DEC	qDEC6	6	22.4–22.8					3.3/−6.5/8.1	
		qDEC7	7	4.8–5.2	5.3/−5.3/9.5		3.8/−5.2/6.8			
		qDEC8.1	8	4.7–5.2		4.3/−6.3/9.5				SSIIIa/Flo5
		qDEC8.3	8	19.9–20.3			4.5/−9.3/11.2			qDEC8a/qDEC-8
		qDEC10	10	13.0–13.1	15.2/−13.7/33.1					qDEC10/qPGC10–2

*GL* grain length, *GW* grain width, *LWR* length to width ratio, *PGWC* percentage of grains with chalkiness, *DEC* degree of endosperm chalkiness

*SZ* Shenzhen, *NJ* Nanjing, *XZ* Xuzhou, *JZ* Jingzhou, and *SY* Sanya. The values of LOD, additive effects, and the phenotypic variance explained of each QTLs were separated by “/”. The additive effects were estimated by the substitution the MH63 allele by the 02428 allele in MH63-ILs and 02428 allele by the MH63 allele in 024283-ILs

*GS3* (Fan et al. 2006); *Chalk5* (Li et al. 2014); *qDEC5a, qDEC8a, qDEC10, qPGWC5a* and *qPGWC8b* (Zhao, 2016); *qDEC-8* (Wan et al. 2005), *qDEC9* (Zhao et al. 2016; Wan et al. 2005); *qPGC5-1, qPGC8-1, qPGC10-2* (Sun et al. 2015); *qPGWC5* (Tan et al. 2000; Li et al. 2009); *qPGWC6* (Tan et al. 2000; Li et al. 2009; Zhao et al. 2016); *qDEC5* (Tan et al. 2000; Li et al. 2009); *GS5* (Li et al. 2011); *GS2* (Hu et al. 2015); *GL2* (Che et al. 2015); *GIF1* (Wang et al. 2008); *OsRab5a* (Wang et al. 2010); *Flo2* (She et al. 2010); *qWB4* (Kobayashi et al. 2013); *SSIIIa* (Fujita et al. 2007); *GL3.1* (Qi et al. 2012; Zhang et al. 2012)

A total of 30 QTL were identified in 02428-ILs, distributing on all 12 chromosomes (Table 3). MH63 alleles were associated with increased GL and LWR at all loci except *qGL4* and *qGL9.2*, and decreased the phenotypic values at all loci for the other traits. A total of 10 (33.3%) QTL distributing on chromosomes 3, 5, 7, 10, 11, and 12 were stably expressed at no less than two environments. *qGL3.1, qGW5.1, qGL7*, and *qLWR7* were identified at four or five locations explaining 5.8–39.1% of phenotypic variances. *qPGWC3, qGL10*, and *qGW12.3* were detected at three locations and explained up to 39.1% variations. Another three QTL (*qPGWC7, qDEC7*, and *qGW11.2*) were detected in two environments.

Among above M-QTL, 9 (14.5%) were detected in both backgrounds, including three for LWR, two for each of GL and DEC, and one of each of GW and PGWC. MH63 alleles at all QTL enhanced AQ, i.e., increased GL, LWR and decreased GW, PGEC, DEC.

### BI- and SE-QTL regions (BISERs) for AQ

M-QTL detected in more than two environments were defined as SE-QTL in this study. Thirteen and ten SE-QTL were detected in each of the reciprocal ILs. Among them, eight QTL (*qGL3.1, qLWR2.1, qGW5.1, qLWR5, qGL7, qLWR7, qPGWC7*, and *qDEC7*) were detected in at least two environments and both backgrounds, designated as BI- and SE-QTL. They were located in four regions on chromosomes 2, 3, 5, and 7. Of the four regions, two affected two or more AQ traits and were defined as BI and SE QTL region (BISER) for AQ. The first BISER (BISER-I) is located in 3.4–3.5 Mb on chromosome 5 harboring two QTL (*qPGWC5* and *qDEC5*) in MH63 background and two QTL (*qGW5.1* and *qLWR5*) in both backgrounds. *qGW5.1* was detected in four environments in each of the reciprocal ILs, explaining 7.3–44.1% of phenotypic variance. *qLWR5* was identified in four and one environments in MH63-ILs and 02428-ILs, respectively, explaining up to 27.4% of phenotypic variance. *qPGWC5* and *qDEC5* were found in four environments and explained 5.4–39.4% of phenotypic variances. BISER-II is located in 4.8–5.2 Mb on chromosome 7. Four QTL (*qGL7, qLWR7, qPGWC7*, and *qDEC7*) were found in at least two environments in MH63-ILs and explained 1.1–23.1% of phenotypic variances. The 02428 alleles decreased GL and LWR while increased PGWC and DEC. *qGL7, qLWR7, qPGWC7*, and *qDEC7* were simultaneously detected in this cluster in 5, 5, 2, and 2 environments in 02428-ILs, respectively, explaining 5.8–13.4% of phenotypic variances. MH63 alleles increased GL and LWR and decreased GW, PGWC, and DEC.

### E-QTL underlying AQ

In MH63-ILs, 7, 1, 5, 6, and 13 digenic epistatic QTL pairs for GL, GW, LWR, PGWC, and DEC were detected across five environments, accounting for 2.7–22.9% of phenotypic variances (Table 4). Among them, nine pairs occurred between two loci without main-effects, three pairs between two M-QTL, and the rest between one M-QTL and one locus. Among them, 20 pairs improved AQ. One pair between the regions of 4.8–5.2 and 10.5–15.3 Mb on chromosome 7 controlling DEC were detected in two environments (SZ and SY), explaining 13.0 and 22.8% of total phenotypic variances, respectively. One pair between the regions of 0–0.3 and 4.2–6.1 Mb on chromosome 9 were found for GW and DEC with 9.7 and 19.9% of phenotypic variances explained, respectively.

**Table 4:** Digenic epistatic QTL pairs (E-QTL) affecting appearance quality traits in the reciprocal ILs derived from MH63 × 02428 and across five locations

ILs	Trait	Loc	Region 1			Region 2			LOD	AA	*R* ^2^ (%)
			Chr	Position (Mb)	M-QTL^a^	Chr	Position (Mb)	M-QTL			
MH63	GL	NJ	1	0.0–1.2		1	12.4–12.7	qGL1.2	5.7	0.17	2.7
		XZ	1	0.0–1.2		3	16.2–17.2	qGL3.1	7.6	−0.42	18.2
			2	19.9–20.4		3	16.2–17.2	qGL3.1	7.5	0.76	18.8
			3	7.0–8.0		3	16.2–17.2	qGL3.1	10.2	0.69	18.5
		JZ	4	7.2–11.3		8	21.3–21.4		5.1	0.23	5.8
			4	7.2–11.3		6	29.9–30.8		5.0	0.25	5.6
			9	11.8–14.0	qGL9.1	11	2.0–2.7		4.3	0.29	6.0
	GW	JZ	9	0.0–0.3		9	4.2–6.1		5.3	−0.09	9.7
	LWR	SZ	3	16.2–17.2	qLWR3.1	5	3.4–3.5	qLWR5	5.6	0.1	9.9
			3	16.2–17.2	qLWR3.1	8	11.0–13.5		5.8	0.1	10.1
		NJ	3	34.9–35.3	qLWR3.2	10	2.3–2.6	qLWR10	5.4	−0.1	3.2
		XZ	1	42.5–42.6		6	24.5–25.8		4.0	−0.2	3.6
			2	0.0–0.4	qLWR2.1	9	14.8–15.1	qLWR9	7.3	0.2	5.7
	PGWC	SZ	1	37.4–37.7		6	2.4–3.2	qPGWC6.1	5.2	−7.6	5.0
		NJ	3	30.0–30.3		5	3.4–3.5	qPGWC5	9.1	−14.7	18.1
			5	3.4–3.5	qPGWC5	12	11.2–12.4		9.4	−12.4	19.0
			7	27.0–27.7		8	9.4–10.6		10.3	−15.6	12.8
		JZ	4	1.1–2.8		4	7.2–11.3	qPGWC4	7.1	−5.9	11.2
			6	2.4–3.2	qPGWC6.1	10	7.0–10.1		7.9	8.1	10.0
	DEC	SZ	3	40.8–53.4		7	4.8–5.2	qDEC7	15.3	7.6	13.3
			4	7.2–11.3		7	4.8–5.2	qDEC7	15.8	8.4	13.0
			7	4.8–5.2	qDEC7	7	10.5–15.3		16.2	7.9	13.0
		NJ	3	30.0–30.3		5	8.5–12.6		7.1	−2.9	3.9
			10	2.5–2.7		10	7.0–10.1		7.7	−3.4	3.4
			5	3.4–3.5	qDEC5	7	10.5–15.3		8.2	5.1	10.6
		XZ	8	9.8–10.2	qDEC8.2	11	27.2–27.4		8.7	19.9	12.0
			8	9.8–10.2	qDEC8.2	12	3.9–5.5		8.8	20.5	22.6
			8	9.8–10.2	qDEC8.2	9	19.9–20.3		8.7	19.5	22.4
			8	9.8–10.2	qDEC8.2	10	4.9–5.4		9.0	19.4	12.0
		SY	9	0.0–0.3		9	4.2–6.1		9.7	−4.9	19.9
			2	27.1–27.8		5	8.5–12.6		10.2	−6.1	22.9
			7	4.8–5.2	qDEC7	7	10.5–15.3		10.9	−9.7	22.8
02428	GL	SZ	1	39.5–40.2	qGL1.3	11	0.0–0.7		4.9	0.26	8.0
		NJ	1	39.5–40.2	qGL1.3	12	15.0–15.1		4.3	0.23	6.2
			4	0.0–0.3		8	14.9–17.4		6.2	0.22	5.5
		XZ	1	3.3–3.8	qGL1.1	11	4.9–5.5		4.0	0.25	8.5
			5	17.2–17.8		11	4.9–5.5		4.3	0.23	8.6
		JZ	1	39.5–40.2	qGL1.3	11	0.0–0.7		4.4	0.27	8.8
			1	39.5–40.2	qGL1.3	12	15.0–15.1		5.4	0.27	7.9
			4	4.8–5.1		9	0.0–0.3		4.2	−0.21	7.6
	GW	SZ	1	30.0–30.2		5	3.4–3.5	qGW5.1	4.0	0.07	9.6
	LWR	NJ	3	22.5–22.6		9	14.8–15.1	qLWR9	4.4	0.1	10.0
		XZ	1	2.4–2.7		4	7.2–11.4	qLWR4.1	4.6	−0.3	20.0
			3	4.9–5.1		5	7.9–13.9		5.2	−0.2	24.9
			4	7.2–11.4	qLWR4.1	7	27.6–27.8		5.8	−0.2	19.2
			4	7.2–11.4	qLWR4.1	8	14.9–17.4		7.8	−0.2	14.4
		JZ	3	2.5–2.7		9	5.0–5.1		3.7	−0.1	10.4
			4	7.2–11.4	qLWR4.1	5	7.9–13.9		3.8	−0.4	21.5
			4	4.8–5.1		6	27.5–27.6		4.0	−0.1	8.1
		SY	4	7.2–11.4	qLWR4.1	7	27.6–27.8		5.0	−0.3	28.1
	PGWC	SZ	4	7.2–11.4	qPGWC4	5	7.9–13.9		5.1	15.4	32.4
		NJ	3	25.0–25.2		9	15.0–15.1		4.5	−9.3	8.9
		XZ	1	14.9–15.5		7	22.4–22.9		5.6	−8.9	8.5
		JZ	1	2.4–2.7		6	2.4–3.2	qPGWC6.1	4.8	−10.2	13.1
			1	19.6–20.4		8	19.9–20.3	qPGWC8	3.8	−11.0	9.1
		SY	7	26.6–27.8		11	10.7–15.5	qPGWC11	3.9	10.1	7.5
	DEC	XZ	1	14.9–15.5		7	22.4–22.9		3.7	−10.0	9.2
		SY	1	12.4–12.7		5	17.2–17.8		3.6	8.8	9.5
			6	7.5–7.8		11	10.7–15.5	qDEC11	4.5	7.5	

*GL* grain length, *GW* grain width, *LWR* length to width ratio, *PGWC* percentage of grains with chalkiness, *DEC* degree of endosperm chalkiness

*SZ* Shenzhen, *NJ* Nanjing, *XZ* Xuzhou, *JZ* Jingzhou, *SY* Sanya

^a^M-QTLs listed in Table 3

In 02428-ILs, 27 epistatic QTL pairs were identified in five environments, each explaining at least 5.5% of phenotypic variation (Table 4). Among them, ten were between two loci without main-effects, the rest between one M-QTL and one locus. 13 E-QTL enhanced AQ. Two pairs between the regions of 39.5–40.2 Mb on chromosome 1 and 0–0.7 Mb on chromosome 11 and between 39.5 and 40.2 Mb on chromosome 1 and 15.0–15.1 Mb on chromosome 12 were detected for GL in two environments (SZ and NJ), explaining 6.2–8.0% of phenotypic variances. A pair between the regions of 7.2–11.4 Mb on chromosome 4 and 27.6–27.8 Mb on chromosome 7 was detected for LWR in XZ and SY. An E-QTL between 7.2 and 11.4 Mb on chromosome 4 and 7.9–13.9 Mb on chromosome 5 were identified for LWR and PGWC, accounting for 21.5 and 32.4% of phenotypic variances. A pair between the region of 14.9–15.5 Mb on chromosome 1 and 22.4–22.9 Mb on chromosome 7 simultaneously decreased PGWC and DEC in XZ, explaining 8.5 and 9.2% of phenotypic variances.

### Validation of BISER-II on chromosome 7

As mentioned above, two BISERs (BISER-I and BISER-II) were detected across five environments in both backgrounds. They were located in 3.4–3.5 Mb on chromosome 5 and 4.8–5.2 Mb on chromosome 7, respectively. There are two cloned genes, *GS5* and *Chalk5* (Li et al. 2011, 2014) in the BISER-I. However, BISER-II was for the first time reported for harboring AQ genes.

**Fig. 3:**
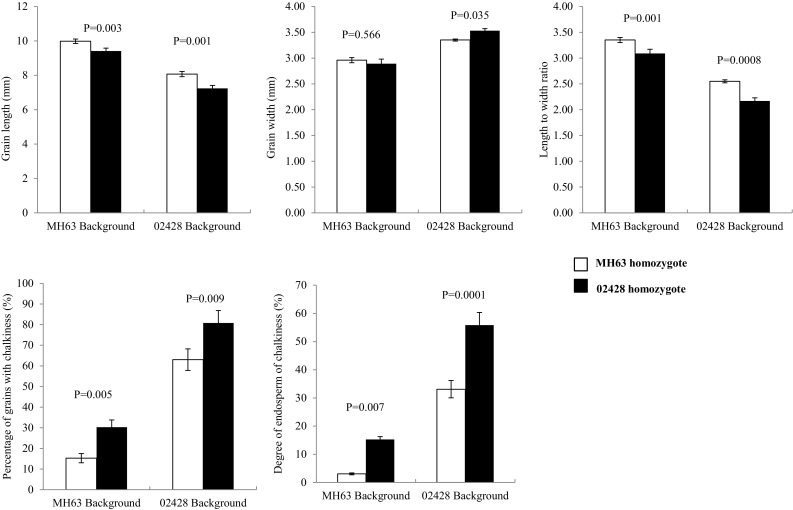
Differences of appearance quality (AQ) traits, including grain length (GL), grain width (GW), length to width ratio (LWR), percentage of grains with chalkiness (PGWC), and degree of endosperm chalkiness (DEC) between MH63 homozygotes and 02428 homozygotes were detected using RM21133 in the two secondary F_2_ populations derived from DQ28 × MH63 in MH63 background and DQ438 × 02428 in 02428 background

To validate BISER-II, two ILs carrying donor fragments in BISER-II, one is DQ28 from MH63-ILs and another is DQ438 from 02428-ILs (Fig. S1), were used for backcrossing to their respective recurrent parent to produce the secondary F_2_ populations. The percentages of recurrent parent genome of the two ILs were 97.46% and 99.01%, respectively. Because most QTL in BISER-II were detected at XZ, so the two secondary F_2_ populations and parents were planted at XZ for measurement of the AQ traits. DQ28 had shorter grains with more chalkiness than MH63, while DQ438 had longer and slender grains with lower chalkiness than 02428 (Table 5). A total of five SSR markers (Table S4) within this region were selected for parental survey, and among them, only RM21133 was found to be polymorphic between the above parents. Using RM21133, all individuals of the two populations were classified into three genotypes: MH63 homozygous, 02428 homozygous, and heterozygous. Plants of two homozygous genotypes were investigated for comparison of AQ traits. The first population (DQ28 × MH63) had 39 plants with MH63 homozygous and 65 with 02428 homozygous. The second population (DQ438 × MH63) had 40 individuals with MH63 homozygous and 56 with 02428 homozygous. The BISER-II showed similar effects on the AQ traits of the two populations (Table 5; Fig. 3). The GL and LWR of MH63 homozygous were significantly higher than that of 02428 homozygous, while MH63 homozygous had lower chalkiness than 02428 homozygous. MH63 homozygous was slender than 02428 homozygous under 02428 and MH63 backgrounds, although the GW values were not significantly different from each other under MH63 background. It was indicated that BISER-II do harbor QTL underlying AQ traits as detected in the reciprocal ILs.

**Table 5:** Differences between DQ28 × MH63 and DQ438 × 02428 F_2_ progenies and their parental lines of appearance quality (AQ) traits at Xuzhou (XZ) location

Lines/genotypes		AQ traits									
		GL (mm)		GW (mm)		LWR		PGWC (%)		DEC (%)	
Parental lines	MH63	9.88 ± 0.08	A	2.89 ± 0.03	A	3.4 ± 0.1	A	10.6 ± 1.9	A	1.8 ± 0.2	A
	DQ28	9.15 ± 0.11	B	2.93 ± 0.05	A	3.1 ± 0.1	B	27.1 ± 2.1	B	16.7 ± 2.5	B
	DQ438	8.23 ± 0.11	C	3.30 ± 0.01	B	2.5 ± 0.0	C	68.3 ± 1.6	C	30.4 ± 6.2	C
	02428	7.51 ± 0.06	D	3.51 ± 0.04	C	2.1 ± 0.0	D	77.5 ± 3.7	D	49.9 ± 7.2	D
DQ28 × MH63 F_2_	MH63 homozygote	9.98 ± 0.13	A	2.96 ± 0.05	A	3.4 ± 0.1	A	15.3 ± 2.2	A	3.1 ± 0.4	A
	02428 homozygote	9.21 ± 0.17	B	2.99 ± 0.09	A	3.1 ± 0.1	B	30.3 ± 3.6	B	15.3 ± 3.0	B
DQ438 × 02428 F_2_	MH63 homozygote	8.17 ± 0.15	C	3.35 ± 0.02	B	2.6 ± 0.0	C	63.0 ± 1.2	C	34.0 ± 9.1	C
	02428 homozygote	7.34 ± 0.17	D	3.53 ± 0.04	B	2.2 ± 0.1	D	80.8 ± 8.1	D	55.8 ± 8.5	D

MH63 and 02428 homozygotes are based on the genotypes of the tightly linked marker RM21133 within the newly identified region for AQ on chromosome 7

Duncan’s grouping at the significant level of *P* ≤ 0.01

*GL* grain length, *GW* grain width, *LWR* length to width ratio, *PGWC* percentage of grains with chalkiness, *DEC* degree of endosperm chalkiness

## Discussion

### QTL for AQ traits

Rice AQ is a very important trait largely affected by both genetic and environmental factors. In this study, a total of 41 and 30 QTL for AQ traits were identified across five environments in the two sets of ILs derived from MH63 and 02428 (Table 3; Fig. 2), respectively. Comparing with the previous studies, most of the grain shape loci were located in the same or adjacent regions of the reported QTL/genes. For instance, *qGL1.2, qGL1.3, qGL7, qGL8*, and *qGL10* for GL, *qGW3, qGW4.1, qGW5.2, qGW7, qGW11.2*, and *qGW12.3* for GW, *qLWR2.2, qLWR3.2*, and *qLWR7* for LWR were mapped to the same or adjacent regions as Bin 1.2, Bin 1.5, *gl7*, Bin 8.4, *qGL10* for GL, Bin 3.7, Bin 4.3, *qGW5c*, Bin 7.4, *qGW11a*, Bin 12.6 for GW, *qLWR2, qLWR3*, and *gs7* for LWR, respectively (Tan et al. 2000; Rabiei et al. 2004; Bai et al. 2010; Zheng et al. 2011; Yan et al. 2014).

However, as for the chalkiness QTL, even though, *qDEC4, qDEC5, qDEC8.2, qDEC8.3, qDEC9*, and *qDEC10* from our report were consistent to *qWB4* (Kobayashi et al. 2013), *qDEC5a* (Zhao et al. 2016)/*qPGC5-1* (Sun et al. 2015)/*Chalk5* (Li et al. 2014)/*qDEC5* (Li et al. 2009; Tan et al. 2000), *qPGC8-1* (Sun et al. 2015), *qDEC8a* (Zhao et al. 2016) /*qDEC-8* (Wan et al. 2005), *qDEC9* (Zhao et al. 2016; Wan et al. 2005), *qDEC10* (Zhao et al. 2016)/*qPGC10-2* (Sun et al. 2015), there still *qDEC6, qDEC7*, and *qDEC11* were reported for the first time. As for the PGWC, *qPGWC5, qPGWC6.1, qPGWC8*, and *qPGWC12* from our report were consistent to *qPGWC5a* (Zhao et al. 2016)/*qPGC5-1* (Sun et al. 2015)/*Chalk5* (Li et al. 2014)/*qPGWC5* (Li et al. 2009; Tan et al. 2000), *qPGWC6* (Li et al. 2009; Tan et al. 2000; Zhao et al. 2016), *qPGWC8b* (Zhao et al. 2016), and *OsRab5a* (Wang et al. 2010), respectively. The *qPGWC3, qPGWC4, qPGWC6.2, qPGWC7*, and *qPGWC11* in our report were novel. In addition, some loci found in this report were also highly consistent to the cloned AQ genes based on the physical positions. For instance, *qGW2* is consistent to *GS2*/*GL2* (Che et al. 2015; Hu et al. 2015) on chromosome 2; the *qGL3.1/qLWR3.1* and *qGL3.2* on chromosome 3 are consistent to *GS3* (Fan et al. 2006; Mao et al. 2010) and *GL3.1* (Qi et al. 2012; Zhang et al. 2012), respectively. On chromosome 4, *qLWR4.2* and *qDEC4* are consistent to *GIF1* (Wang et al. 2008) and *Flo2* (She et al. 2010), respectively. *qGL5, qGW5.1, qLWR5, qPGWC5*, and *qDEC5* are clustering surrounding the region of *Chalk5* (Li et al. 2014) and *GS5* (Li et al. 2011). The *qDEC8.1* on chromosome 8 and *qLWR12.2* on chromosome 12 were mapped together with the *SSIIIa/Flo5* (Fujita et al. 2007) and *OsRab5a* (Wang et al. 2010), respectively. In total, 30% of the ten DEC loci and 55.6% of the nine PGWC loci are novel in our report. Allelism of the above AQ QTL detected in this study to the previously reported QTL/genes is worthy of further clarification after fine mapping and cloning of the AQ QTL.

### BI-QTL for AQ traits

There are significant effects of genetic background on complex traits including the AQ traits, which have largely lagged behind the application of the identified QTL/genes in the rice molecular breeding. The consistency of the QTL among different genetic backgrounds are relatively low for complex traits such as salt tolerance (15.4%) (Cheng et al. 2011; Qiu et al. 2015), drought tolerance (17.9%) (Wang et al. 2013), sheath blight resistance (18.2%) (Xie et al. 2008), and even for grain yield components (21%) (Mei et al. 2006). In present study, only 9 out of the 62 (14.5%) AQ QTL were commonly detected in both backgrounds which is in agreement with the above reports on other traits using the reciprocal ILs derived from Teqing and Lemont. Additionally, epistatic QTL are more sensitive to genetic background (Liao et al. 2001). In this work, there is no E-QTL commonly detected in both genetic backgrounds for grain chalkiness, which is consistent to the previous reports on panicle number (Liao et al. 2001), sheath blight resistance (Xie et al. 2008) and salt tolerance (Cheng et al. 2011). Moreover, the fact that there are still a few pairs of epistasis for AQ traits detected across environments other than between different backgrounds indicated that the genetic background has stronger affect on AQ traits than the environmental factors, especially for PGWC and DEC with lower heritability. This can also explain why there are significant effects of genetic background on AQ traits (Kobayashi et al. 2013). Thus, breeders must pay much attention especially when QTL information applied in molecular breeding for AQ traits, as genetic backgrounds were different between mapping and breeding populations.

### SE-QTL for AQ traits

Another bottleneck for the improvement of complex traits is the environmental sensitivity of the QTL detected, especially for the AQ traits. When we took together the three reports (Wan et al. 2005, 2006; Zhao et al. 2016) working on the AQ traits through different environments, we found that about 60.4% of the AQ were stably detected across different environments, including 41.2% for grain shape and 62.9% for grain chalkiness. However, in different reports, the ratio of SE-QTL for grain chalkiness varied largely, from 36.4% (Wan et al. 2005) to 84.8% (Zhao et al. 2016). In present study, 17 SE QTL out of 62 QTL (27.4%) for AQ were identified across five environments; the portion of SE-QTL for the grain shape (17.7%) and grain chalkiness (9.7%) were similar. Most of the SE-QTL for AQ traits were harboring the known gene/QTL. For example, the SE-QTL *qLWR5* and *qGL7/qLWR7* were stably expressed under all five environments. The *qGL3.1* consistent to *GS3* (Fan et al. 2006) was stably expressed under four environments. It is true for *qGW5.1*/*qPGWC5.1*/*qDEC5* which were consistent to *GS5*/*Chalk5* (Li et al. 2011, 2014). These SE-QTL would be very useful resources for the molecular breeding on AQ traits.

### Useful BISERs for AQ improvement

In present study, two regions harboring BI- and SE-QTL affecting most AQ traits were detected across five environments using the reciprocal ILs derived from MH63 and 02428. They were BISER-I (3.4–3.5 Mb on chromosome 5) and BISER-II (4.8–5.2 Mb on chromosome 7). The MH63 alleles at both BISERs enhanced AQ. The BISER-I harbored many BI- and SE-QTL for AQ traits detected in this study and was also detected for grain chalkiness in two recombinant inbred lines derived from Teqing and Lemont across nine environments (Zhao et al. 2016), containing two cloned genes, *Chalk5* and *GS5. GS5* encodes putative serine carboxypeptidase (Li et al. 2011). MH63 belongs to H94 haplotype (http://ricevarmap.ncpgr.cn/), while 02428 (named as 2428 in the database) belong to Zhenshan 97 haplotype which was significantly wider than H94 haplotype. *Chalk5* encodes a vacuolar H^+^-translocating pyrophosphatase with inorganic pyrophosphate hydrolysis (Li et al. 2014). Sequence analysis divided rice cultivars into seven haplotypes based on the nucleotide polymorphisms of Zhenshan 97 and H97, and they were placed into two classes: haplotype 1–4 in class A and haplotype 5–7 in class B. Class A had higher *Chalk5* expression and chalkiness than class B. MH63 belonged to type 5, which was significantly lower than type 1 including 02428. We have also compared the sequences of MH63 and 02428 throughout the BISER-I (3,390,000–3,470,000 bp) on chromosome 5. We found that the two parents (MH63 and 02428) shared a very high sequence identity of 96.78% throughout the BISER-I region; however, when we focused on the sub-regions of *GS5* (3,439,210–3,443,769) and *Chalk5* (3,335,248–3,339,817), the sequence identities dramatically decreased to only 61.46 and 60.61%, respectively. As for the promoter region controlling the *Chalk5* function, the sequences of MH63 and 02428 of *Chalk5* have been found to belong to H94 and ZS97 haplotypes, respectively (Li et al. 2014). Thus, *GS5* and *Chalk5* are the possible candidate genes of the BISER-I affecting AQ.

Another region, BISER-II, harboring BI- and SE-QTL for most AQ traits except GW detected in this study was located on the short arm of chromosome 7. Up to date, *qPGWC-7* is the only reported locus on chromosome 7 controlling grain chalkiness. It was located at the end of the long arm on chromosome 7 detected by PA64 × 9311 CSSLs (Zhou et al. 2009). Thus, the BISER-II is a new region. MH63 alleles at this region increased the grain length but decreased the grain width and grain chalkiness simultaneously in both genetic backgrounds (Table 3; Fig. 2). By adopting the favorable alleles within the BISER-II, rice breeders can acquire satisfactory AQ by positive selections on slender grain shape. This is also consistent to the common senses of breeders working on conventional rice breeding.

However, it is also notable that even within these two BISERs, in comparing to the grain shape QTL, the chalkiness QTL were still relatively sensitive to both the genetic background and environmental effects. For example, in BISER-I, *qPGWC5* and *qDEC5* expressed under only MH63 background throughout all environments except for XZ, the northern most of the five locations; interestingly, as for BISER-II *qPGWC7* expressed throughout two environments (SZ and XZ) in both genetic backgrounds, and *qDEC7* expressed throughout three environments (SZ, XZ, and SY) in MH63 background and throughout two environments (SZ and XZ) in 02428 background. For verification of the BISER-II, we planted the validation materials at one of the above two locations, XZ, belonging to the *indica*/*japonica* mix-cultivating area at the down stream of the Yangtze River. Our confirmation work at XZ has approved that our mapping work for BISER-II is reliable. This strongly indicated the usefulness of the MH63 alleles in BISER-II which can be adopted by breeders for the improvement of the AQ traits at least in XZ similar areas.

Taking together, BISER-I and BISER-II are two important regions with favorable alleles from slender grain varieties, so pyramiding of favorable alleles at QTL in the two regions from slender grain rice will be most likely to much improve AQ traits for rice variety especially for hybrid rice by MAS.

#### Author contribution statement

XQ, KC, WL, XO, YZ, LY, FF, JY carried out phenotyping and genotyping. DX, JX, and ZL managed the project. XQ and TZ analysed the data. XQ, TZ, JX wrote the paper.

## Electronic supplementary material

Below is the link to the electronic supplementary material.

Supplementary material 1 (PPT 192 KB)

Supplementary material 2 (DOC 131 KB)
