# Supplementary material for: Examining two sets of introgression lines reveals background-independent and stably expressed QTL that improve grain appearance quality in rice (Oryza sativa L.)
Source: Theor Appl Genet. 2017 Mar 15;130(5):951–67. doi: 10.1007/s00122-017-2862-z (PMC5395602; doi:10.1007/s00122-017-2862-z)
Supplement: Supplementary file 1 — Supplementary material 1 (PPT 192 KB) [file 122_2017_2862_MOESM1_ESM.ppt]

## Slide 1
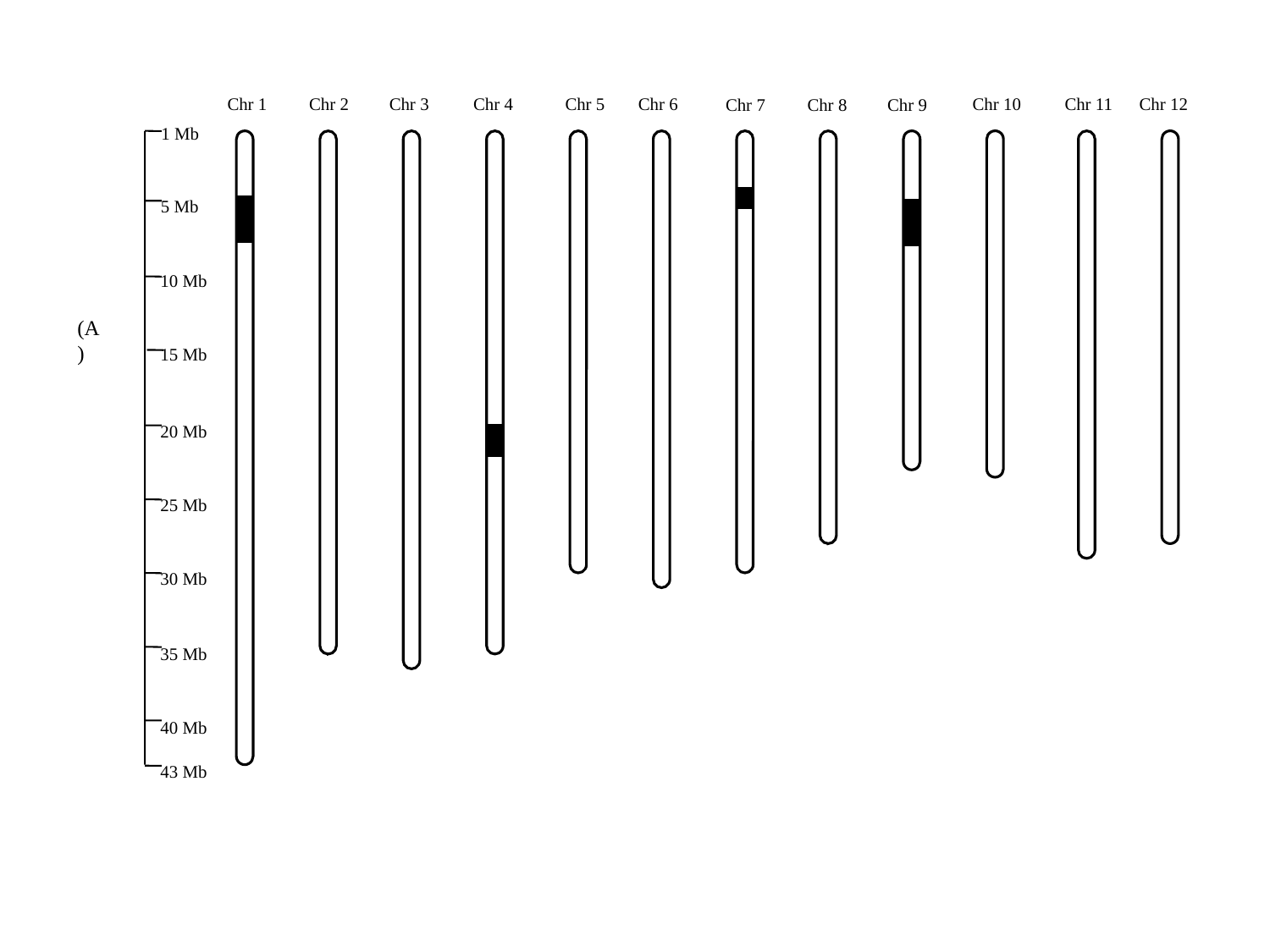

Chr 1
Chr 2
Chr 3
Chr 4
Chr 5
Chr 6
Chr 10
Chr 11
Chr 12
Chr 7
Chr 8
Chr 9
1 Mb
5 Mb
10 Mb
15 Mb
20 Mb
25 Mb
30 Mb
35 Mb
40 Mb
43 Mb
(A)

## Slide 2
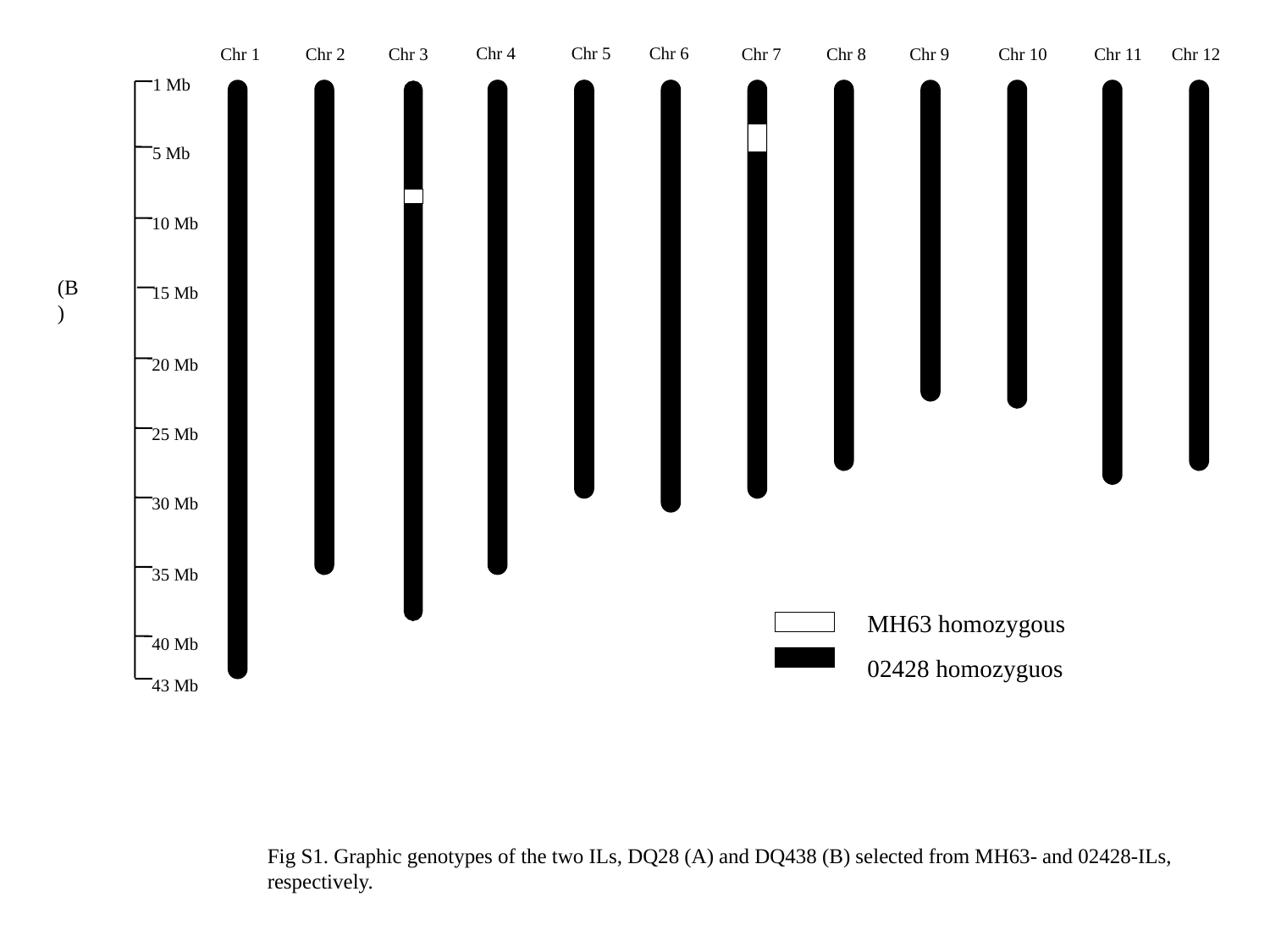

Chr 4
Chr 5
Chr 6
Chr 1
Chr 2
Chr 3
Chr 7
Chr 8
Chr 9
Chr 10
Chr 11
Chr 12
1 Mb
5 Mb
10 Mb
15 Mb
20 Mb
25 Mb
30 Mb
35 Mb
40 Mb
43 Mb
(B)
MH63 homozygous
02428 homozyguos
Fig S1. Graphic genotypes of the two ILs, DQ28 (A) and DQ438 (B) selected from MH63- and 02428-ILs, respectively.
