# Supplementary material for: Examining two sets of introgression lines reveals background-independent and stably expressed QTL that improve grain appearance quality in rice (Oryza sativa L.)
Source: Theor Appl Genet. 2017 Mar 15;130(5):951–67. doi: 10.1007/s00122-017-2862-z (PMC5395602; doi:10.1007/s00122-017-2862-z)
Supplement: Supplementary file 2 — Supplementary material 2 (DOC 131 KB) [file 122_2017_2862_MOESM2_ESM.doc]

**Table S1 Thresholds from 1000 permutations adopted in the M-QTL detection for the five appearance traits in ILs of two backgrounds at 5** locations.

| **Traits ^a^** | **Loc ^b^** | **MH63-IL** | **02428-IL** |
| --- | --- | --- | --- |
| GL | SZ | 2.99 | 4.13 |
|  | NJ | 2.87 | 4.25 |
|  | XZ | 2.83 | 4.01 |
|  | JZ | 2.95 | 3.89 |
|  | SY | 2.81 | 3.05 |
| GW | SZ | 2.86 | 3.88 |
|  | NJ | 2.75 | 3.79 |
|  | XZ | 2.66 | 3.65 |
|  | JZ | 2.88 | 3.21 |
|  | SY | 2.86 | 3.08 |
| LWR | SZ | 3.01 | 3.33 |
|  | NJ | 3.13 | 3.58 |
|  | XZ | 3.15 | 3.11 |
|  | JZ | 2.99 | 3.05 |
|  | SY | 2.84 | 3.23 |
| PGWC | SZ | 2.66 | 2.89 |
|  | NJ | 2.57 | 2.78 |
|  | XZ | 2.59 | 2.66 |
|  | JZ | 2.61 | 2.89 |
|  | SY | 2.53 | 2.53 |
| DEC | SZ | 2.78 | 3.28 |
|  | NJ | 2.65 | 3.05 |
|  | XZ | 2.83 | 3.36 |
|  | JZ | 2.96 | 3.24 |
|  | SY | 2.88 | 3.51 |

1. GL = grain length, GW = grain width, LWR = length to width ratio, PGWC = percentage of grains with chalkiness, and DEC = degree of endosperm chalkiness.
2. SZ = Shenzhen, NJ = Nanjing, XZ = Xuzhou, JZ = Jingzhou, and SY = Sanya.

**Table S2 Correlation coefficients of appearance quality traits estimated in the two sets of reciprocal IL populations derived from MH63 × 02428 at 5 locations.**

| **Traits ^a^** | **Loc ^b^** | **GL** | **GW** | **LWR** | **PGWC** | **DEC** |
| --- | --- | --- | --- | --- | --- | --- |
| GL | SZ |  | -0.34^****^ | 0.86^****^ | -0.42^****^ | -0.41^****^. |
|  | NJ |  | -0.29^****^ | 0.84^****^ | -0.27^****^ | -0.24^***^ |
|  | XZ |  | -0.31^****^ | 0.86^****^ | -0.38^****^ | -0.42^****^ |
|  | JZ |  | -0.28^****^ | 0.84^****^ | -0.31^****^ | -0.28^****^ |
|  | SY |  | -0.38^****^ | 0.87^****^ | -0.21^***^ | -0.21^***^ |
| GW | SZ | -0.08 |  | -0.76^****^ | 0.43^****^ | 0.46^****^ |
|  | NJ | -0.25^****^ |  | -0.76^****^ | 0.46^****^ | 0.47^****^ |
|  | XZ | -0.27^****^ |  | -0.75^****^ | 0.37^****^ | 0.44^****^ |
|  | JZ | -0.16^*^ |  | -0.75^****^ | 0.53^****^ | 0.52^****^ |
|  | SY | -0.23^****^ |  | -0.78^****^ | 0.50^****^ | 0.52^****^ |
| LWR | SZ | 0.77^****^ | -0.69^****^ |  | -0.52^****^ | -0.52^****^ |
|  | NJ | 0.82^****^ | -0.75^****^ |  | -0.44^****^ | -0.43^****^ |
|  | XZ | 0.85^****^ | -0.73^****^ |  | -0.48^****^ | -0.54^****^ |
|  | JZ | 0.21^***^ | -0.69^****^ |  | -0.51^****^ | -0.47^****^ |
|  | SY | 0.82^****^ | -0.75^****^ |  | -0.41^****^ | -0.41^****^ |
| PGWC | SZ | -0.19^***^ | 0.49^****^ | -0.44^****^ |  | 0.92^****^ |
|  | NJ | -0.34^****^ | 0.63^****^ | -0.58^****^ |  | 0.86^****^ |
|  | XZ | -0.30^****^ | 0.43^****^ | -0.46^****^ |  | 0.92^****^ |
|  | JZ | -0.25^****^ | 0.48^****^ | -0.17^*^ |  | 0.93^****^ |
|  | SY | -0.20^***^ | 0.62^****^ | -0.49^****^ |  | 0.91^****^ |
| DEC | SZ | -0.21^***^ | 0.47^****^ | -0.44^****^ | 0.92^****^ |  |
|  | NJ | -0.44^****^ | 0.57^****^ | -0.61^****^ | 0.90^****^ |  |
|  | XZ | -0.44^****^ | 0.51^****^ | -0.59^****^ | 0.89^****^ |  |
|  | JZ | -0.28^****^ | 0.48^****^ | -0.17^**^ | 0.96^****^ |  |
|  | SY | -0.28^****^ | 0.60^****^ | -0.52^****^ | 0.92^****^ |  |

1. GL = grain length, GW = grain width, LWR = length to width ratio, PGWC = percentage of grains with chalkiness, and DEC = degree of endosperm chalkiness.
2. SZ = Shenzhen, NJ = Nanjing, XZ = Xuzhou, JZ = Jingzhou, and SY = Sanya.
3. Data under and above the diagonal are correlation coefficients in MH63-ILs and 02428-ILs, respectively. ^*^, ^**^ , ^***^ , and ^****^ indicate the significant level of P ≤ 0.05, 0.01, 0.005, and 0.001 levels, respectively.

**Table S3 Correlation coefficients between five locations for the appearance quality traits in the reciprocal IL populations derived from MH63 × 02428.**

| **Loc ^a^** | **Traits ^b^** | **SZ** | **NJ** | **XZ** | **JZ** | **SY** |
| --- | --- | --- | --- | --- | --- | --- |
| SZ | GL |  | 0.90^****^ | 0.89^****^ | 0.83^****^ | 0.81^****^ |
|  | GW |  | 0.79^****^ | 0.79^****^ | 0.75^****^ | 0.86^****^ |
|  | LWR |  | 0.80^****^ | 0.90^****^ | 0.80^****^ | 0.90^****^ |
|  | PGWC |  | 0.38^****^ | 0.43^****^ | 0.41^****^ | 0.41^****^ |
|  | DEC |  | 0.48^****^ | 0.48^****^ | 0.45^****^ | 0.54^****^ |
| NJ | GL | 0.75^****^ |  | 0.92^****^ | 0.78^****^ | 0.80^****^ |
|  | GW | 0.58^****^ |  | 0.88^****^ | 0.68^****^ | 0.79^****^ |
|  | LWR | 0.68^****^ |  | 0.95^****^ | 0.75^****^ | 0.86^****^ |
|  | PGWC | 0.53^****^ |  | 0.38^****^ | 0.35^****^ | 0.35^****^ |
|  | DEC | 0.42^****^ |  | 0.48^****^ | 0.46^****^ | 0.46^****^ |
| XZ | GL | 0.77^****^ | 0.93^****^ |  | 0.75^****^ | 0.81^****^ |
|  | GW | 0.60^****^ | 0.85^****^ |  | 0.69^****^ | 0.78^****^ |
|  | LWR | 0.70^****^ | 0.92^****^ |  | 0.73^****^ | 0.87^****^ |
|  | PGWC | 0.59^****^ | 0.55^****^ |  | 0.21^***^ | 0.28^****^ |
|  | DEC | 0.54^****^ | 0.66^****^ |  | 0.29^****^ | 0.35^****^ |
| JZ | GL | 0.86^****^ | 0.69^****^ | 0.69^****^ |  | 0.69^****^ |
|  | GW | 0.60^****^ | 0.41^****^ | 0.42^****^ |  | 0.67^****^ |
|  | LWR | 0.22^***^ | 0.13 | 0.17^*^ |  | 0.73^****^ |
|  | PGWC | 0.68^****^ | 0.54^****^ | 0.53^****^ |  | 0.32^****^ |
|  | DEC | 0.67^****^ | 0.43^****^ | 0.54^****^ |  | 0.41^****^ |
| SY | GL | 0.90^****^ | 0.72^****^ | 0.73^****^ | 0.80^****^ |  |
|  | GW | 0.67^****^ | 0.55^****^ | 0.60^****^ | 0.61^****^ |  |
|  | LWR | 0.86^****^ | 0.65^****^ | 0.70^****^ | 0.26^****^ |  |
|  | PGWC | 0.60^****^ | 0.47^****^ | 0.49^****^ | 0.56^****^ |  |
|  | DEC | 0.55^****^ | 0.54^****^ | 0.50^****^ | 0.56^****^ |  |

1. SZ = Shenzhen, NJ = Nanjing, XZ = Xuzhou, JZ = Jingzhou, and SY = Sanya.
2. GL = grain length, GW = grain width, LWR = length to width ratio, PGWC = percentage of grains with chalkiness, and DEC = degree of endosperm chalkiness..
3. Data under and above the diagonal are correlation coefficients in MH63-ILs and 02428-ILs, respectively. ^*^, ^**^ , ^***^ , and ^****^ indicate the significant level of P ≤ 0.05, 0.01, 0.005, and 0.001 levels, respectively.

**Table S4 Primers used for validation of BISE-II on chromosome 7.**

| **Name** | **Chr.** | **PhyPos** | **Primer_forward** | **Primer_reverse** |
| --- | --- | --- | --- | --- |
| RM21128 | 7 | 4,854,256 | CTTCCACCTCAGTGAAAGGATGG | CGTGTGATTCCCAGTATGATTGC |
| RM21129 | 7 | 4,894,816 | CGTGAGGTAGACCACGCTTCTGG | TACACAGGGATTACACAGGGATTTGG |
| RM21131 | 7 | 4,927,820 | ATATAGGGCAGCAAAGGCTGTGG | CCTCCAGATTGTCACCCTTCTCG |
| RM21132 | 7 | 4,928,177 | CGTCTACTACCACTACCACTCAGC | GCTAGGTAGACCAAGCTCTCG |
| **RM21133** | **7** | **4,928,209** | **GCTTCCTCGAGGGATGGTACGG** | **TCCGAGACCTTGGCCATAGACG** |
